# Hay Fever

**Published:** 1885-07

**Authors:** 


**Hay Fever.** By Charles E. Sajous, M. D., Instructor of Rhinology and Laryngology in Jefferson Medical College, President Philadelphia Laryngological Society, &c. Illustrated with thirteen wood engravings. F. A. Davis, publisher. No. 1217 Filbert street. 1885.

This little book advocates the treatment of hay fever by superficial organic alteration of the nasal mucous membrane. The success of the treatment is illustrated by numerous cases. Dr. Sajous has admirably presented the subject, and as this method of treatment is now generally recognized as efficient, we can recommend this book to all physicians who are called upon to treat this troublesome disorder.

